# Influence of Stage Cooling Method on Pore Architecture of Biomimetic Alginate Scaffolds

**DOI:** 10.1038/s41598-017-16024-x

**Published:** 2017-11-23

**Authors:** Yuanming Zhang, Conger Wang, Wei Jiang, Wenqian Zuo, Guangting Han

**Affiliations:** 1 Laboratory of New Fiber Materials and Modern Textile, The Growing Base for State Key Laboratory, Qingdao University, Qingdao, 266071 P.R. China; 2 College of Textiles and Fashion, Qingdao University, Qingdao, 266000 P.R. China

## Abstract

Porous scaffold is widely used in the field of tissue engineering. However, the anisotropic structure of actual extracellular matrix (ECM) of human tissue pose a challenge to the scaffold structure that pore size should be changed in gradient. Here we report a stage cooling method to fabricate alginate scaffold with gradient pores. Eight cooling models were set according to different temperature steps, different initial temperature, and different time duration. The thermal characterization of solution during cooling process were recorded and scaffold morphology were observed. The results revealed that the temperature steps mainly affected pore shape, while the initial temperature and time duration mainly affected pore size. By altering the initial temperature and time duration, scaffold exhibited cellular and gradually enlarged pores on the vertical axial direction (10–65 μm at base, 50–141 μm at top). With this stage cooling method, pore shape and pore size could be easily tailored and scaffold with gradient structure could be fabricated.

## Introduction

Tissue engineering has been widely used for reconstruction of damaged tissue or organs, such as artificial skin, synthetic bone and vascular grafts. A promising approach in tissue engineering is to design and fabricate biodegradable scaffolds with ideal pores structure suitable for cell adhesion, growth and proliferation^1^. Every human tissue and organ has its own specific three-dimensional (3D) extracellular matrix (ECM) structure. Cells in a 3D support for tissue engineering typically align new ECM components according to the inner specific architecture of the bioscaffold^2–4^. The actual ECM of human tissue typically exhibited anisotropic structure with gradient pores, e.g. human skin has enlarged pore size with distance away from the skin surface. Scaffold with gradient pore structure ranging from 25 μm to 120 μm is supposed to be suited for ingrowth of skin tissue^5^. Therefore, tissue engineering scaffold with a specific gradient pore structure resembling the actual ECM would have great potential for the regeneration and repair of a broad range of damaged anisotropic tissues.

Alginate is a naturally biodegradable polymer that typically obtained from seaweeds. It has been certificated by Food and Drug Administration (FDA) for tissue engineering application. With similar chemical component to ECM, combined with its biocompatibility and hydrophilicity, alginate is favorable for cell adhesion and growth^6–9^. The composite materials of alginate with chitosan, collagen or polyving alcohol have been applied to the research of tissue repairing scaffold^10,11^.

Various approaches have been reported to date for the production of porous scaffolds. For example, rapid prototype (RP)^12^, injection moulding and solvent evaporation^13^, porogen leaching^14,15^, and gas foaming^16,17^. Such methods may be effectively applied for synthetic polymers but are not to be recommended for alginate, since it is neither molten nor insoluble in most solvents. Hence, despite the success of the above mentioned methods in producing anisotropic scaffolds, the processes are complexity. On the other hand, the use of additional chemicals in the fabrication process is not environmentally friendly.

Freeze-drying method is an important technique to overcome the above obstacles. In this case the solution or slurry of polymer is frozen, thereby creating an interpenetrating network of ice crystals^18,19^. These ice crystals are then removed by reducing the chamber pressure to induce sublimation, thus leading to the formation of a porous scaffold. This method is applied to natural polymers like alginate and collagen^20,21^. Wang^22^ fabricated a gradient scaffold by stacking three freezing-dried porous collagen membranes layer by layer to form a “sandwich” scaffold, which exhibited significantly faster wound closure effect than homogeneous scaffolds. Zhang^23^ mixed ice particulates into collagen solutions. The mixtures were stacked together and freeze dried to form scaffolds with gradient pores. By these two methods, layers with different pore diameter were stacked together and there exists visible demarcation on the junction. However, the ideal scaffold was expected to have gradually changed pore size.

By altering the freezing conditions, such as freezing temperature, freezing time and the freezing moulds, pore size and shape could be tailored^24^. Davidenko *et al*.^25^ designed five freezing moulds to achieve different heat transfer rate within solution. By confining temperature gradient on horizontal and vertical direction, collagen scaffold with both circled and aligned pores with different diameter were produced. Pawelec *et al*. confined temperature gradient on the axial direction by set different solution height^26^. With increase of solution height, pores at top of scaffold becoming bigger and pillared structure, arranging from 90 to 170 μm.

Manipulation of alginate scaffold structure would be a matter of great interest, since this would provide an intrinsically biocompatible way of tailoring the scaffold applying to the precise requirements of tissue regeneration. With this in mind, the current work was directed to the influence of stage cooling process upon the inner structures of alginate scaffolds, aiming to prepare scaffolds resembling 3D organization of the skin ECM. By adopting different temperature step, different initial temperature and different cooling time duration, scaffold with anisotropic pores gradient distributed along the vertical direction was produced. The concept of stage cooling is new and simple, and the pore size and distribution range could be predicted.

## Materials and Methods

### Scaffold fabrication

**Figure 1:**
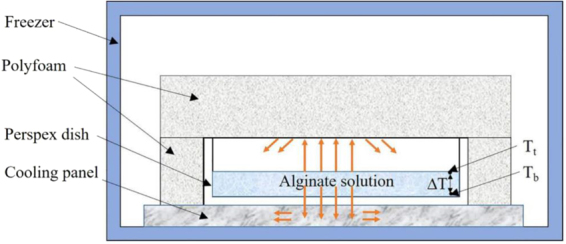
Cross-section of the perspex dish coated by polyfoam with solution in it. The heat conduction was confined on the vertical axial direction with T_t_ > T_b_, with T_t_ the temperature at top of solution, and T_b_ the temperature at base of solution. The temperature gradient within solution on vertical direction was labeled as ∆T.

Sodium alginate powders (M_W_ = 300,000, M_w_/M_n_ = 1.5, Qingdao Hyzlin Biology Development Co., Ltd., China) was dissolved by water to prepare solution with concentration of 1.5%. The solution was homogenized at 15,000 rpm for 3 h at room temperature. After standing at 10 °C for 48 hours to remove bubbles, 12 g of the solution was poured into perspex dish with inside diameter of 64 mm and height of 12 mm, the height of solution was about 5 mm. The perspex dish was then put into an ultra-low temperature freezer with bottom placed on the cooling panel and the wall and top were coated by heat-insulated polyfoam as shown in Fig. 1. Thus, heat transfer between solution and environment were limited only downward through bottom of the dish and temperature gradient (∆T) on vertical direction within solution was generated.

In order to study the influence of stage cooling to scaffold microstructure, eight cooling models were set up as shown in Table 1. Model A was stage cooled from initial temperature of −75 °C, experienced 5 steps to the final temperature of −15 °C. Model B and C went through less steps from −75 °C to −15 °C, 3 and 2 steps separately. Model D and E both went through 5 steps, but different initial and final temperature. Model D was cooled from −60 °C to −5 °C, while Model E from −90 °C to −30 °C. Model F, G and H all cooled from −75 °C to −15 °C, but the time duration at each step was different.

**Table 1:** Stage cooling models.

Temperature (°C) Time (min) Model	−90	−75	−60	−45	−30	−15	−5
A	/	10	8	6	4	480	/
B	/	18	/	10	/	480	/
C	/	28	/	/	/	480	/
D	/	/	10	8	6	4	480
E	10	8	6	4	480	/	/
F	/	12	10	8	6	480	/
G	/	8	6	4	2	480	/
H	/	6	4	2	/	480	/

After cooling process, the frozen solution was lyophilized in freeze drier (LGJ-IO, Beijing Songyuan Huaxing Technology Develop Co., Ltd., China) at 0 °C for 24 hours under vacuum at less than 1 Pa. As the sodium alginate scaffold was soluble, it need to be cross-linked to get dissoluble calcium alginate scaffold. The first-degree samples were immersed into CaCl_2_ solution with concentration of 5% for 1 hour at room temperature and then flushed with distilled water. After freezing for 12 hours at −30 °C and secondary freeze-drying process (0 °C under vacuum at less than 1 Pa for 24 hours), the final calcium alginate scaffolds with gradient pores were prepared. Five samples for each model was fabricated.

### Thermal characterization

Thermocouples were used to measure the temperature at the base and top of solution every minute during cooling process, recorded as T_b_ and T_t_ separately. The crystallization temperature (°C), the moment when ice crystallization occur (min), cooling rate (°C/min), and the biggest temperature difference between top and base of solution (∆T_max_, °C) could be obtained from temperature curves.

### Scaffold morphology

Scaffold morphology was characterized by scanning electron microscopy (JSM-6390LV, JOEL, Japan). The cross section from top to base of the scaffold was observed to identify the gradient pore structure. Pore length and height was measured by Image-Pro Plus to calculate average pore size by Eq. (1) (Shapiro & Cohen, 1997).

1 $${\rm{d}}=\sqrt{l\times h}$$

In which, *d* is the average pore size, mm; *l* is pore length, mm; *h* is pore height, mm.

## Results and Discussion

### Thermal characterization

**Figure 2:**
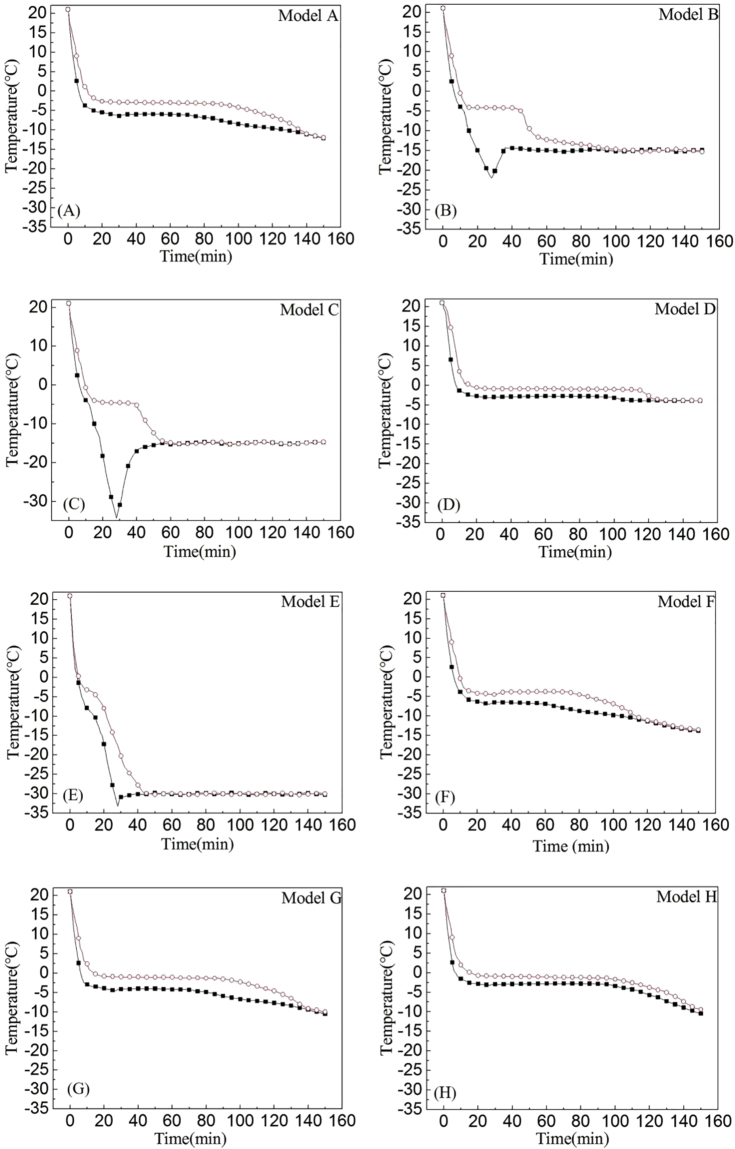
Temperature curves of eight models during cooling process. The squares indicate temperature at base of solution, and circles indicate top of solution.

The temperature curves of eight models during cooling process were showed in Fig. 2, and the characteristic values were listed in Table 2.

**Table 2:** The temperature characteristic values of eight cooling models.

Model	∆T_max_ (°C)	Crystallization temperature (°C)		Moment of crystallization (min)		Cooling rate (°C/min)	
		Top	Base	Top	Base	Top	Base
A	4.30	−2.65	−5.30	20	16	−1.18	−1.46
B	17.83	−4.10	−6.50	17	13	−1.67	−2.12
C	29.71	−4.80	−6.50	15	13	−1.72	−2.12
D	3.50	−0.75	−2.73	23	18	−0.95	−1.32
E	15.60	−4.50	−7.90	15	10	−1.70	−2.89
F	4.40	−3.90	−6.10	18	17	−1.38	−1.59
G	4.20	−0.85	−3.60	21	17	−1.04	−1.45
H	2.20	−0.80	−2.73	22	17	−0.99	−1.36

#### Model A

In this model, it was found from Fig. 2(A) that the temperature at base layer of solution quickly decreased with cooling rate of −1.46 °C/min, while the cooling rate of top layer was −1.18 °C/min. Then at the 16^th^ minute, the cooling curve at base of solution flattens at −5.3 °C. This period was called latent heat section, during which the ice crystals began to form and grow in the entire solution. But crystallization at top layer of solution was delayed till the 20^th^ minute at −2.65 °C because of the heat transfer direction within solution were limited only downward. Due to the hysteresis of heat conduction, the temperature gradient was produced between top and base layers of solution, and at some point reached ∆T_max_≈4.3 °C. When the whole solution was totally frozen, the temperature at both top and the base layers of solution slowly decreased approaching to −15 °C with time.

#### Model B and C

As shown in Fig. 2(B,C), the cooling curves of these two models resemble each other in shape, but the thermal characterizations varied greatly. Model B, during the first cooling step at −75 °C, the base layer of solution started phase transmission at the 13^th^ minute. Because of the fast cooling rate (−2.12 °C/min), the crystallization heat could be quickly dissipated with no measurable platform, and temperature at base continuously decreased till the 28^th^ minute when it rose approaching to the final environment temperature (−15 °C). The heat transfer at upper layer of solution was lagged behind with lower cooling rate of −1.67 °C/min, but still started crystallizing at the first cooling stage of −75 °C. As shown in Table 2, the crystallization on top layer of Model B started at the 17^th^ minute with crystallization temperature of −4.1 °C. During the cooling process, ∆T_max_ within solution was 17.83 °C. Compared with model B, Model C has faster cooling rate on upper layer of solution (−1.72 °C/min) and lower crystallization temperature (−4.8 °C), and it behaved the largest ∆T_max_ among 8 models (29.71 °C). Taken model A together with these two models, it was found that the different freezing step effected the thermal characterization of models during freezing process.

#### Model D and E

With the highest initial temperature among these eight models (−60 °C), Model D displayed the slowest cooling rate (−1.32 °C/min at base and −0.95 °C/min at top), shown in Fig. 2(D). The base of solution started crystallizing at −2.73 °C on the 18^th^ minute, nearly at end of the second cooling stage. While the top layer started freezing at −0.75 °C on the 23^rd^ minute, at the end of the third cooling stage, and the ∆T_max_ was only 3.5 °C. On the contrary, with the lowest initial temperature (−90 °C), it can be seen from Fig. 1(E) that Model E exhibited the quickest cooling rate (−2.89 °C/min at base and –1.7 °C/min at top) and the lowest crystallization temperature among these 8 models (−7.9 °C at base and −4.5 °C at top) with ∆T_max_ of 15.6 °C. The crystallization of base layer started at the first cooling stage on the 10^th^ minute, while the top layer started at the second stage on the 15^th^ minute. Taken model A together with these two models, it was found that the different initial temperature effected the thermal characterization of models during freezing process.

#### Model F to H

Compared to model A, model F, G and H all shared similar temperature curves with little difference (Fig. 2(F–H)). With the shortening of time at each cooling stage, the cooling rate in these three models decreased in sequence (Model F: −1.59 °C/min at base and −1.38 °C/min at top; Model G: −1.45 °C/min at base and −1.04 °C/min at top; Model H: −1.36 °C/min at base and −0.99 °C/min at top), while the crystallization temperature increased (Model F: −6.1 °C at base and −3.9 °C at top; Model G: −3.6 °C at base and −0.85 °C at top; Model H: −2.73 °C at base and −0.8 °C at top). Crystallization on base layer of Model F started at the middle of the second cooling stage, while Model G at the end of the third stage, and Model H at the fourth stage. The ∆T_max_ of Model F and G was similar (Model F: 4.4 °C; Model G: 4.2 °C), while Model H was the smallest (2.2 °C). From model F, G and H, It can be seen that the different time duration effected the thermal characterization of models during freezing process, but when the time duration of stage is shortened, the effect of stage cooling is weakened.

### Scaffold morphology

**Figure 3:**
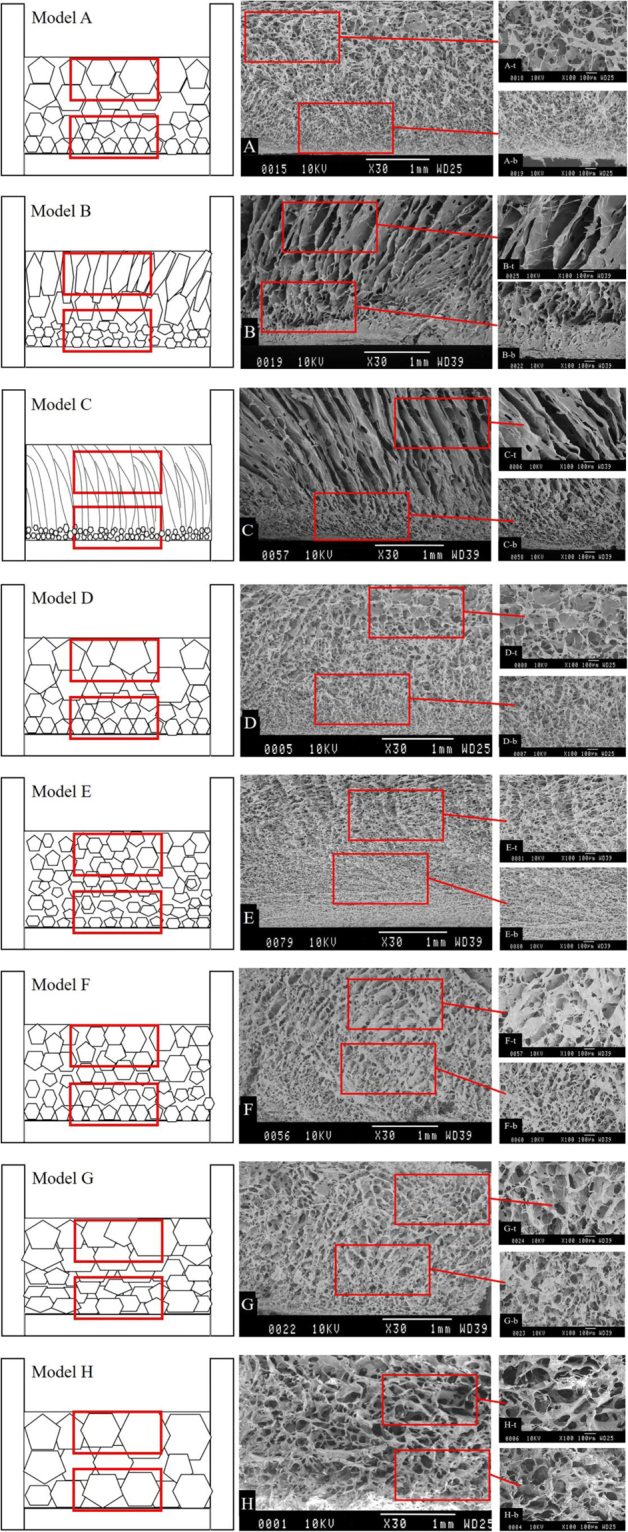
SEM images of scaffolds from eight cooling models.

The alginate scaffold prepared from the eight models exhibited morphology with much difference. Figure 3 showed the SEM graphs of vertical axial cross-section and Table 3 showed the pore size and wall thickness of these scaffolds, the values are means of five samples.

**Table 3:** Pore size of scaffolds from eight cooling models.

Model	Pore size (μm ± SD)		Wall thickness (μm ± SD)	
	Top	Base	Top	Base
A	100.0 ± 15.0	20.0 ± 3.5	22.0 ± 3.4	9.6 ± 2.7
B	/	15.0 ± 2.1	42.6 ± 6.2	18.3 ± 2.1
C	/	8.0 ± 1.5	57.1 ± 8.6	23.4 ± 2.4
D	141.0 ± 20.2	25.0 ± 2.4	23.2 ± 2.9	9.1 ± 1.4
E	50.0 ± 11.1	10.0 ± 1.8	14.7 ± 1.2	7.9 ± 1.6
F	70.0 ± 12.6	18.7 ± 1.6	21.7 ± 5.1	10.2 ± 3.1
G	122.0 ± 22.4	65.0 ± 4.7	26.8 ± 1.9	19.7 ± 2.2
H	137.0 ± 12.1	130.0 ± 18.3	30.4 ± 2.8	24.4 ± 2.8

#### Model A

In this model, as described previously, the moderate temperature gradient was produced between the top and the base of the frozen sample and the ∆T_max_ was 4.3 °C (Fig. 2(A) and Table 2). As the temperature gradient is small, ice crystals were able to grow freely at all directions. This would lead to the ice crystals growing with different rate from the base layer to the upper layer, inducing to the formation, after sublimation, of an anisotropic distribution of rounded pores on scaffold with gradually enlarged diameters from base to top. Confirmation of this assumption was made by SEM examination of the scaffold produced in the model (Fig. 3(A)), where longitudinal sections displayed gradient pores structure, with small pores at base (20 ± 3.5 μm), and gradually bigger toward top layer (100 ± 15.6 μm). All pores were approximate cellular shaped with good connectivity and thin walls (9.6 ± 2.7 μm at base and 22.0 ± 3.4 μm at top) between neighboring pores. The diameter of pores at base section of scaffold were suitable for the construction of epidermal cells with its compact structure, while the larger pores at top section were suited for the adhesion of dermal cells.

On the bottom area, the cooling rate is high and the temperature is low so that ice crystals grew faster. As a consequence, the pores resulting from sublimation of ice crystals on this surface area is smaller. In the upper region, however, with lower cooling rate and higher temperature, the growth rate of ice crystals is slower, hence the pore size was larger compared with that observed at base.

#### Model B and C

SEM images of the longitudinal sections of the alginate matrices produced in model B and C are shown in Fig. 3(B,C). It is interesting to note that lamellar structure alignment along the temperature gradient is only observed in the longitudinal top segment (Fig. 2(B-t,C-t)), while in its matching bottom segment (Fig. 2(B-b,C-b)) tiny pores (scaffold B: 15 ± 2.1 μm; scaffold C: 8 ± 1.5 μm) were presented. The wall thickness between neighboring pores are much larger than that of Model A, and the wall between lamellar pores at top layer of scaffold are much thicker than cellular pores at the base section.

As can be seen from Fig. 2(B,C), these two models created a greater temperature gradient between the top and the bottom layer of solution compared with model A, and ∆T_max_ of model B and C was 17.83 °C and 29.71 °C (Table 1) respectively. The larger temperature gradient providing greater driving force on the heat transfer direction for the growth of ice crystals. Ice crystals grow faster on the vertical direction but slower on horizontal, induced to a defined lamellar structure alignment with thick walls in the upper half of the two samples. While at the base section, the lower cooling temperature and faster cooling rate induced to smaller and round ice crystals, which left uniform and small pores at base of scaffold after sublimation.

#### Model D and E

As expected, Scaffolds D and E have cellular gradient pores similar to scaffold A (Fig. 3(D,E)). Model D tend to form bigger and less pores (25 ± 2.4 μm at base and 141 ± 20.2 μm at top) with similar pore walls (9.1 ± 1.4 μm at base and 23.2 ± 2.9 μm at top), while scaffold E have much smaller pores (10 ± 1.8 μm at base and 50 ± 11.1 μm at top) and thinner pore walls (7.9 ± 1.6 μm at base and 14.7 ± 1.2 μm at top). Comparing with Model A, it could be seen that the initial temperature effected pore size on the top layer greater than the base layer, and the size difference between base and top became smaller with lower initial temperature.

With initial temperature of −60 °C, Model D was moved to environment of −5 °C at the 29^th^ minute. This provided a slow enough cooling rate and loose of driving force for ice crystals. Ice crystals at top layer of solution were very instable with its crystallization temperature of −0.75 °C. The instable ice crystals melted and refroze ceaselessly, developing into much bigger crystals and widened the pore size variation of scaffold on the axial direction (25~141 μm). The range of pore size of scaffold D is approaching to the ECM of human skin, with pores at top section a little larger. With lower initial temperature of −75 °C, scaffold from Model A got smaller pores and narrowed pore size variation (20~100 μm). Model E was just opposite to Model D, with its initial temperature of −90 °C, it behaved the fastest cooling rate among these eight models. Both top and base layer of solution were frozen quickly without apparent platform period (Fig. 2(E)). Stable ice crystals were formed and the scaffold pore size were further reduced (10~50 μm).

#### Model F to H

In accordance with expectation, Scaffold F and G displayed cellular gradient pores (Fig. 3(F,G)), while pores in scaffold H take the shape of homogeneous structure (Fig. 3(H)). Comparing with scaffold A, pores at top layer of scaffold F was smaller (70 ± 12.6 μm), while the base layer was similar (18.7 ± 1.6 μm). Then with the decrease of time duration at each temperature step, pores become bigger both at top and base of scaffold. However, the time duration effected pore size on the base layer greater than the top layer, with size difference declined. Scaffold G have pores of 65 ± 4.7 μm at base and 122 ± 22.4 μm at top, while scaffold H contains cellular pores of similar size, 130 ± 18.3 μm at base and 137 ± 12.1 μm at top. When the time duration of each cooling stage shortened, the wall thickness slightly increased, but the difference between base and top section was narrowed.

With the shortening of time duration at each cooling stage, the cooling rate was decreased and crystallization temperature was increased, inducing bigger pores and smaller size gradient. The crystallization at base layer of solution all started from the 17^th^ minute, but at different cooling stage. Especially for Model H, with its crystallization started at the fourth cooling stage, most of its cooling process were under the constant temperature of −15 °C. The effect of stage cooling is no more significant, which provided a nearly uniform temperature within the solution. The released heat of crystallization melts small and allow big crystals to grow freely at all directions, melting and rebinding with adjacent ones. The pores were much bigger than other samples and tending to be isotropic (with pore size of 130–137 μm, and wall thickness of 24.4–34 μm).

The explanations given above for the pore profiles observed in the longitudinal sections at the top and the bottom of the freeze-dried samples are in complete concordance with the well-known and widely documented^4,18,24,27,28^ temperature dependence of scaffold pore size produced by lyophilization technique. It is generally accepted that lower cooling temperatures yield smaller pore sizes for matrices.

The human skin has functionally gradient pore structure range 25~120 μm. In this current work, advances have been made in tailoring pore structure on scaffold with pore size that changed gradually, and Scaffold A (20~100 μm) and D (25~141 μm) generally meet the size requirements. It could be deduced that when the initial temperature was set between −60 °C to −75 °C, pore size could be accuracy controlled. By controlling the parameters of stage cooling, more porous scaffolds with complex shape and size gradient could be fabricated for application of various tissue therapies.

## Conclusion

In this study, freeze-drying method with stage cooling process was adopted to produce scaffolds with gradient pores mimicking the gradient structure of human skin. By varying the cooling temperature steps, initial environmental temperature and time duration, the thermal characterization within solution was discrepant, further inducing the change of pore shape and size on scaffold. The temperature steps mainly affected pore shape, and scaffold showed pillared pores at upper layer when the step was enlarged above 30 °C. The initial cooling temperature and time duration mainly affected pore size on scaffold. The pores at base of scaffold was smaller, corresponding to the dermal layer of skin; while pores at top of scaffold was larger, well suited for the growth of epidermal cells. Scaffold A and D generally meet the size requirements of 25~120 μm, and with the initial temperature between −60 °C to −75 °C, pore size rang was predicted to be accuracy controlled. By this easy and cheap method of stage cooling, scaffold pore size and shape could be tailored for potential usage of more tissues with gradient architecture.
